# Dynamics of essential interaction between firms on financial reports

**DOI:** 10.1371/journal.pone.0225853

**Published:** 2019-12-18

**Authors:** Hayato Goto, Eduardo Viegas, Hideki Takayasu, Misako Takayasu, Henrik Jeldtoft Jensen

**Affiliations:** 1 Centre for Complexity Science and Department of Mathematics, Imperial College London, London, United Kingdom; 2 Institute of Innovative Research, Tokyo Institute of Technology, Yokohama, Japan; 3 Sony Computer Science Laboratories, Tokyo, Japan; RIKEN, JAPAN

## Abstract

Companies tend to publish financial reports in order to articulate strategies, disclose key performance measurements as well as summarise the complex relationships with external stakeholders as a result of their business activities. Therefore, any major changes to business models or key relationships will be naturally reflected within these documents, albeit in an unstructured manner. In this research, we automatically scan through a large and rich database, containing over 400,000 reports of companies in Japan, in order to generate structured sets of data that capture the essential features, interactions and resulting relationships among these firms. In doing so, we generate a citation type network where we empirically observe that node creation, annihilation and link rewiring to be the dominant processes driving its structure and formation. These processes prompt the network to rapidly evolve, with over a quarter of the interactions between firms being altered within every single calendar year. In order to confirm our empirical observations and to highlight and replicate the essential dynamics of each of the three processes separately, we borrow inspiration from ecosystems and evolutionary theory. Specifically, we construct a network evolutionary model where we adapt and incorporate the concept of fitness within our numerical analysis to be a proxy real measure of a company’s importance. By making use of parameters estimated from the real data, we find that our model reliably replicates degree distributions and motif formations of the citation network, and therefore reproducing both macro as well as micro, local level, structural features. This is done with the exception of the real frequency of bidirectional links, which are primarily formed as a result of an entirely separate and distinct process, namely the equity investments from one company into another.

## Introduction

Recent developments in the field of complexity science have led to a renewed interest in social and economic activities [1–3] being captured as complex systems characterised properties such as small world [4] and scale-free [5]. It has previously been observed that business firm networks belong to a class of the complex systems interacting with others in distinct ways, accompanied by specific scaling relations [6–15].

A typical example of business firm networks is the inter-firm business transactions network, whereby nodes are firms that link through business transactions from customers and suppliers, producing a directional money flow (with the opposite direction being the goods/service flows). Recent studies have found that such type of network contains the generic properties similar to other complex networks, however, with particular formation dynamics. As a result, models have been proposed to understand the dynamics of the networks [11, 16–20] beyond the generic formation models. Here, we highlight the work carried out by Takayasu et al. [11]. The investigation into the Japanese inter-firm business transactions networks, including over 1 million firms in Japan, and the subsequent follow on work carried out by Miura et al. [18]. Within the latter, a simple business network model—whereby a directed link connecting nodes represents money flows between a pair of firms—is proposed in order to evaluate the effects of new establishments, bankruptcies, and mergers and acquisitions (M&As). Each of these businesses processes is separately represented—respectively—by creation, removal and aggregation of nodes and related links. It follows that such model accurately reproduced the statistical characteristics of the inter-firm business transactions networks. The dataset underpinning the above research was provided by one of the leading corporate research bureaus in Japan. It encompasses information on business trading partners—such as customers and suppliers—of almost all corporate ecosystem within Japan, with both public as well as private firms. The dataset contains detailed information of circa 85% of all registered companies within the country that make up 98% of total annual sales [15].

Additional related research provides insight into the concept of ‘metabolism’ of firms [15, 18]. It shows that the cumulative distribution of the age of firms, since establishment, is well characterised by an exponential function, *exp*(−*t*/*τ*). The *τ* ∼ 55 years is the characteristic decay time so that it is assumed that a firm disappears randomly following a Poisson process. Likewise, the distribution of the business transactions is also approximated well by the exponential function with the *τ* ∼ 6 years [21]. Therefore, only about 1.8% of nodes and 16.7% of links on the inter-firm business transactions networks rewire over the following year. In other words, these characteristics indicate that most components of the inter-firm business transactions network are stable.

Furthermore, Atalay et al. [16] proposed a scale-free model and confirmed this model using yearly firm-level buyer-supplier networks of the US economy, covering a total of over 39,000 firm-year observations from 1979 to 2007. The dataset used for the research was compiled by one of the credit rating agencies, which had lists of core partners of all the listed firms and a few private firms, generated by company information such as financial reports. In general, public firms are required to disclose all audited financial statements by regulatory listing requirements. Almost all firms usually refer their essential information such as business partners and capital ties in their financial reports to explain prospects of their businesses to external stakeholders [22, 23]. Hence, it can be expected that the changes to the business models and fundamental relationships are reflected within the yearly financial reports. Therefore, the essential dynamics of interactions among firms can be derived from the financial reports. However, this potentially differs from the specific dynamics of the inter-firm business transactions networks earlier described, as the latter only provides information related to major transactions whereas the former also contains middle sized as well as smaller business transactions. In any case, little is currently known and researched about differences between these two network dynamics due to the lack of data related to smaller private firms’ financial reports.

In summary, although a network solely derived from financial information is only partial, it is found to replicate the key structural features of the wider real interfirm business network, with more specific differences reconciled by a relatively simple evolutionary dynamic algorithm [24].

Motivated by these discussions, a key objective of this study is to investigate the dynamics of firms interactions derived from the financial reports and how they interrelate to the real business transaction networks. Here, we make use of a comprehensive dataset of Japanese firms’ financial reports collected by one of the largest corporate research providers in Japan. This dataset covers not only listed firms but also small and middle-scaled private firms in Japan, covering about 400, 000 firms, so that this is large enough to compare with the previous studies as we described above. To do this, inter-firm business citations networks are compiled by text information in the financial reports (hereafter referred to as “citation networks”) written about one’s business summary given “Line of Business”, “Characteristics of the Company”, “Operating Performance”, “Financial Position and Fund-Raising Capacity”, and “Latest Trend and Prospects” [25].

Essentially, we find that about quarter of links on the citation network are exchanged for next year so that the rewiring is one of the dominant processes in the network, even though only about 16.7% links on the inter-firm business transactions network exchanges for next year [21]. In addition, we propose a network generation model with fitness, which is a real number measuring companies’ importance proposed by Caldarelli et al. [26], and conduct numerical analysis by our model with parameters estimated by real data. We verify that our model is able to replicate major statistical characteristics of the citation network, with the exception of the real frequency of bidirectional links that are mainly generated by inter-firm investment relationships. This result suggests that investment interactions tend to be formed as a result of a different, and separate, process from other business transactions.

## Materials and methods

### Empirical data analysis

#### About dataset

In this report, we analyse Japanese firm dataset provided by Teikoku Databank Ltd (TDB), one of the largest corporate research providers in Japan. It is common business practice within Japan for companies to gather detailed corporate information from business partners in order to build long term trustworthy relationships as well as to manage credit and operational risk. The data collection process and credit analysis are normally outsourced and carried out by professional third-party organisations such as TDB, one of the largest corporate research providers in Japan, that has been assessing the credit status of firms for 119 years. TDB’s credit research reports include detailed information about the financial statements of firms, their history profile, business partners, management structure as well as banking transactions and relationships. Companies are tracked over time by an allocated unique IDs, “Teikoku Company Code”, including all private enterprises, business owners, government organizations and other public offices in Japan. This code is embedded in all databases so that one can combine all different types of data provided by TDB with ease.

Table 1 shows details of three specific datasets, namely (a) financial reports, containing detailed financial data from around 400, 000 firms including the years 2017 and 2018; (b) inter-firm business transactions records for about 800, 000 firms, which has been extensively used in academic research over the last fifteen years [11, 12, 15, 18, 20, 21, 27]; and (c) investment relationships form business partners, groups companies, capital ties and structures as well as other investment relationships. The later data is consistent with that found in the first database as firms usually report such essential information within their financial statements. Therefore, the real network of investment relationships and business partners combined with the inter-firm business transactions can be used to directly compare with the citation network derived from the scanning of the financial reports.

**Table 1:** Details of the database.

Dataset	Financial reports	List of inter-firm business transactions					
Year	Number of reporting	Number of firms	Number of links	Min	Max	Mean	Median
2015	392,822	734,127	4,869,600	1	12,117	13.3	6
2016	389,131	756,950	5,088,622	1	12,601	13.5	6
2017	390,727	786,717	5,475,568	1	13,116	13.9	6
2018	389,294	810,627	5,789,700	1	13,678	14.3	7

Dataset	List of inter-firm investment relationships					
Year	Number of firms	Number of links	Min	Max	Mean	Median
2015	265,832	408,615	1	1,926	3.1	2
2016	273,033	425,880	1	1,996	3.2	2
2017	279,961	443,409	1	2,097	3.2	2
2018	286,118	459,337	1	2,199	3.3	2

#### Scaling relations

The citation network is constructed by the scanning of text data in the financial reports automatically. This is therefore unstructured, which essentially leads to the construction of some incorrect links due to companies having the same names or common noun names. For example, there is a restaurant run by CHINA Co., Ltd. in Japan. Even though this company is very small, it gets a large number of citations from companies who traded with China as country since it is one of the most important destinations for their exports.

To eliminate such errors, we attempt to detect outliers where the total number of citations is inconsistent with the size of the company. In order to set a standard in one’s number of citations, we observe a scaling relation between a link number on the citation network *k*_*c*_ and a sum of link numbers on both the inter-firm business transactions and the inter-firm investment network *k*_*b*_ (hereafter referred to as “business partners”) as the measure of firm size. Besides, this is in similar manner with previous study [24] and it can be substituted by number of employees or annual sales, given that there are scaling relations among those quantities as described in [12].

We next produce an intersection network among the inter-firm business transactions network, the inter-firm investment network, and the citation network. This is composed of overlapping links between the citation network and the rest of the networks so that it is logical to regard the intersection network as a subset of the citation network without data harvesting induced any errors. Therefore, the intersection network can be used to observe real scaling relation between *k*_*b*_ and *k*_*c*_.

**Fig 1:**
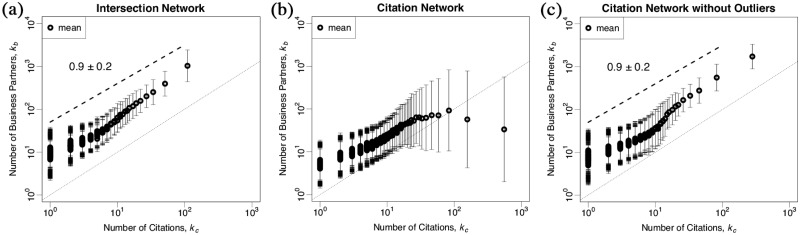
Scaling relations between a mean of pairs of *k*_*b*_ and *k*_*c*_ on each type of network in a log-log scale plot. Each panel (a), (b), and (c) shows the relation on the intersection network, the citation network, and the citation network without the outliers, respectively. There is a scaling relation such that $k_{b}\propto k_{c}^{0.9\pm 0.2}$ on both panels (a) and (c) though the relation collapse in range of large *k*_*c*_ on panel (c).

Fig 1 shows scaling relations between the mean of pairs of *k*_*b*_ and *k*_*c*_ on each type of network. We here divide *k*_*c*_ into $\sqrt{N}$ bins—i.e. square-root choice -, where *N* is total number of firms on each network and each bin contains the same number of items, and we follow on by calculating the mean of *k*_*b*_ per bin. Panel (a) shows the relation on the intersection network. We have identified there is a scaling relation such that $k_{b}\propto k_{c}^{0.9\pm 0.2}$ since the intersection network is a subset of the citation network without any errors as mentioned before. On the other hands, panel (b) shows the relation on the citation network. In a range of large *k*_*c*_, this collapses the relation we found in panel (a). Considering this, we delete firms that deviate from the scaling relation $k_{b}\propto k_{c}^{0.9\pm 0.2}$. Concretely, we eliminate a firm which has *k*_*c*_ > *μ* + 2*σ* for each range of *k*_*b*_ to get the same scaling relation on the citation network as that on the intersection network as shown in panel (c). In the following discussion, we use the citation network without the outliers.

Besides, Table 2 shows number of nodes and links in each type of network. This shows that 94,009 firms with 1,035,482 links on the citation network have been eliminated by this procedure in 2018. However, only 2% of all nodes on the intersection network have been eliminated corroborating the fact that almost all valid data is preserved. The elimination rate is also very consistent with the implied error rate arising from the manual procedure described in. Furthermore, in order to obtain additional comfort with regards to the effectiveness of the filtering method, we have manually sampled 1,000 firms within our dataset in the descending order of the respective differences between *k*_*b*_ and *k*_*c*_. We found that links were correctly removed from 989 firms within the sample, and therefore, implying an error rate of around 1.1% which as perfectly acceptable for the purposes of this study. We also noted that all financial reports that we have made use of have proofread manually since TDB has strict data checking process to maintain data quality. Therefore, abbreviations or misspellings of companies are rare events within the reports. Moreover, we conduct segmentation of sentences into their parts of speech and extract only noun words by MeCab, which is an open-source text segmentation library for use with text written in the Japanese language [28]. We then replace any misspelt word or abbreviation with the correct word through this process so that the rare cases are reduced even further and incorrect links caused by such human error are eliminated.

**Table 2:** Details of each type of network.

Type	Intersection network					
Year	Number of firms	Number of links	Min	Max	Mean	Median
2015	226,430	296,835	1	16	1.7	1
2016	233,571	313,343	1	13	1.8	1
2017	254,378	363,399	1	14	1.9	1
2018	264,835	386,065	1	19	1.9	1
Type	Citation network					
Year	Number of firms	Number of links	Min	Max	Mean	Median
2015	460,401	1,487,213	1	44	4.0	3
2016	458,541	1,516,814	1	33	4.1	4
2017	460,772	1,593,223	1	33	4.3	4
2018	460,867	1,622,695	1	43	4.4	4
Type	Citation network without outliers					
Year	Number of firms	Number of links	Min	Max	Mean	Median
2015	347,344	496,722	1	17	2.0	2
2016	350,980	521,062	1	17	2.1	2
2017	364,146	576,105	1	17	2.2	2
2018	366,858	587,213	1	22	2.2	2

#### Metabolism of network evolution

It has previously been revealed that a model, structured by the processes of node creation, annihilation and coagulation, together with a preferential attachment rule—where new nodes are attached to older ones with a probability which is a growing function of the number of pre-existing links [5, 29]—reproduces a degree distribution that follows a power law consistent with that of Japanese inter-firm business transactions network [18, 27]. Therefore, the balance between new entrants, bankruptcies and mergers plays the key role in the time evolution of this network. In terms of ‘metabolism’ of the network, the cumulative distributions of both node lifespan and link lifespan on the inter-firm business transactions network are well characterised by an exponential function, *exp*(−*t*/*τ*), where *τ* is the characteristic decay time [18, 21]. Concretely, *τ* ∼ 55 years for nodes and *τ* ∼ 6 years for links so that about 1.8% of nodes and 16.7% of links on the networks exchanges for next year, respectively.

Similar to previous studies on the interfirm business network, we also observe here the occurrence probabilities from 2015 to 2018 for both nodes and links to evaluate the specific dynamics of the citation network. It is assumed that the citation network is a non-growing network because a number of newcomers is the almost same amount as that of disappears, though about 14.9% of nodes disappeared for each year in Table 3. Additionally, the citation network has minimal numbers of coagulation of nodes—i.e. mergers—in comparison with the inter-firm business transactions network.

**Table 3:** Number of firms on the citation networks by type from 2017 to 2018.

Year	Number of firms	Newcomers	Disappears	Absorbed
2015	347,344	59,393	56,516	220
2016	350,980	60,335	56,521	178
2017	364,146	67,446	54,104	176
2018	366,858	57,385	54,483	190

Table 4 shows the details of occurrence probabilities for links. This also indicates that the network seems to be a non-growing network in terms of links, though the characteristics are different from links on the business transactions network. Links account for about quarter of the citation network are exchanged for next year so that it appears that rewiring is one of the dominant processes in the citation network, being time independent.

**Table 4:** Number of links on the citation networks by type from 2015 to 2018.

Type of link	Type of process	From 2015 to 2016		From 2016 to 2017		From 2017 to 2018	
		Number of links	Ratio	Number of links	Ratio	Number of links	Ratio
Stay	-	362,070	-	385,537	-	430,776	-
New links	Link creation	53,601	18.3%	73,261	22.5%	61,332	20.3%
	Node creation	105,391	35.9%	117,307	36.0%	95,105	31.5%
Dead links	Link annihilation	45,119	15.4%	49,479	15.2%	54,541	18.1%
	Node annihilation	89,533	30.5%	86,046	26.4%	90,788	30.1%

#### Asymmetrical degree distribution

Data from several countries suggest that firm indicators such as annual sales, number of employees, and the number of business partners follow a power law [6–11]. In particular, it has been reported that both a number of in- and out-links (i.e. in- and out-degrees) on the inter-firm business transactions network in Japan follows a power law with a cumulative exponent 1.4 ± 0.1 [11]. In contrast, we find that in- and out-degrees are asymmetrical in the citation network as follows.

**Fig 2:**
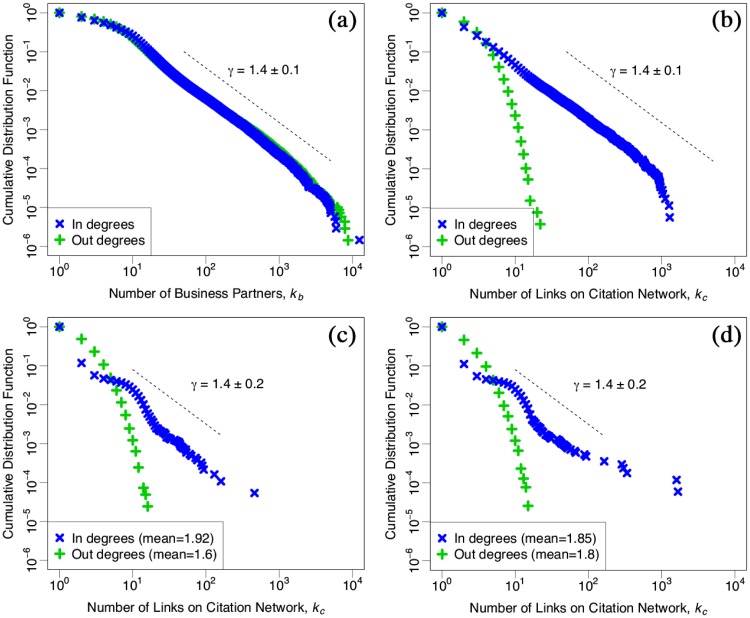
Cumulative distributions of in- and out-degree in 2018 in a log-log scale plot. Each panel (a) and (b) shows this distribution on the inter-firm business transactions network and on the citation network, respectively. Each green plus-mark and blue cross shows out-degrees and in-degrees, respectively. Each panel (c) and (d) shows this distribution generated by newcomers and disappears on the citation network, respectively. Each mean number of in-degrees is about 1.92 in panel (c) and 1.85 in panel (d), respectively, and each mean number of out-degrees is about 1.60 in panel (c) and 1.80 in panel (d), respectively.

Fig 2 shows cumulative in- and out-degree distributions for 2018 in a logarithm scaled plot. Each panel (a) and (b) shows this distribution on the inter-firm business transactions network *k*_*b*_ and on the citation network *k*_*c*_, respectively, and each green plus-mark and blue cross shows out-degrees and in-degrees, respectively. As reported previously, in panel (a), both the distributions of in- and out-degree of *k*_*b*_ follow a power law with a cumulative exponent *γ* = 1.4 ± 0.1. By contrast, panel (b) shows that an out-degree distribution of *k*_*c*_ exponentially decays, though an in-degree distribution follows a power law with an exponent *γ* = 1.4 ± 0.1 the same as the distribution of the number of business transactions in panel (a). The reason is presented as follows: out-degrees on the citation network, i.e. a number of citing a name of one’s business partners have upper bounds because the size of the financial reports is limited. Meanwhile, in-degrees on the citation network are generated by the number of citations from other firms’ financial reports so that the in-links mainly concentrates in large-scale firms.

Furthermore, Fig 2(c) and 2(d) show the cumulative degree distributions for newcomers and disappears. Both in-degree distributions follow the power law with the exponent *γ* = 1.4 ± 0.2 the same as panel (b). Besides, each mean number of the newcomers and the disappears is about 1.92 and 1.85 for in-degrees and about 1.6 and 1.8 for out-degrees, respectively.

#### Network motif

**Fig 3:**
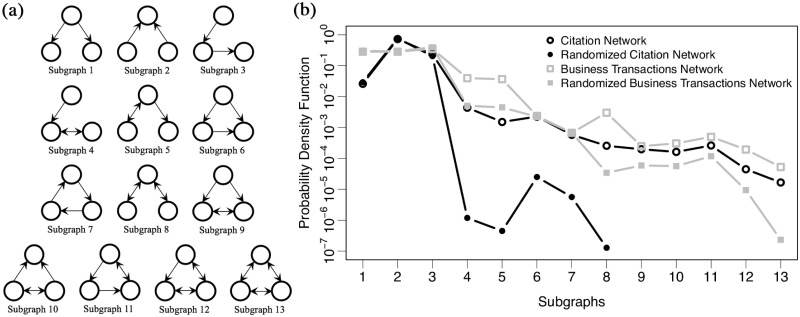
Network motifs on both the citation network and the inter-firm business transactions network in 2018. Each panel (a) and (b) shows all 13 types of three-node connected subgraphs and a probability density distribution of the subgraphs, respectively. Each black circle, black filled circle, grey square, and grey filled square shows the probability distribution of the subgraphs on the citation network, that on the randomised network by the citation network initially, that on inter-firm business transactions network, and that on the randomised network by inter-firm business transactions network initially, respectively.

Motif formation is one of the basic characteristics of interactions among three-node subsets in networks [30, 31]. To observe microscopic characteristics of the citation network, we observe the motif formation distribution and compare this with that of the business transactions network. Moreover, we randomise both the business transactions network and the citation network over 10, 000, 000 times while preserving firms’ degrees [32] (hereinafter, this is called “randomised network”) and observe the motif formation distributions. Each panel (a) and (b) in Fig 3 shows all 13 types of three-node connected subgraphs and a probability density distribution of the subgraphs, respectively. Each black circle, black filled circle, grey square, and grey filled square in panel (b) shows the probability distribution of the subgraphs on the citation network, that on the randomised network based on the citation network, that on inter-firm business transactions network, and that on the randomised network based on inter-firm business transactions network, respectively.

It appears that subgraph number two is dominant within the citation network, whereas subgraphs number one, two, and three are dominant in the business transactions network, as also reported in previous studies [33, 34]. By comparison with the result by the randomised network on the inter-firm business transactions network, there are differences in frequencies of subgraphs that have bidirectional links such as number four, five and seven to thirteen. Consequently, it seems that the occurrence probability of the bidirectional links on the citation network is unusually high. Moreover, there are significant differences in frequencies of all subgraphs within the citation network, except for the dominant subgraphs. This indicates that, in terms of network motif, therefore, the dynamics of the citation network is far from random. Besides, one can assume that the difference of probabilities between subgraph one and two on the citation network might be caused by the in-out asymmetry since both the probabilities on the business transaction network, which does not have the asymmetry, are almost the same.

## Results and discussion

### Model analysis

#### Fitness

Various models that generate different types of complex networks have been proposed. As described above, Miura et al. [18] make use of a simple business network model in which a directed link connecting nodes represents money flow between a pair of firms, and they took into account the effects of new establishments, bankruptcies, and mergers and acquisitions (M&As) by creation of new nodes, removal of nodes, and aggregation of nodes together with links, respectively. Additionally, by using a merger kernel estimated through an M&A data analysis, the model reproduces business network characteristics with the parameters estimated by real firm data [15, 27]. As shown in Tables 3 and 4, however, the citation network has a few numbers of M&As but a large number of rewirings. Reka et al. [35] proposed an evolving network model that was taken into account the effects of link additions, rewiring, and node additions. In terms of the total number of nodes, however, this also does not fit with the citation network because this is not an evolving network but a quasi-steady-state network, and additionally, the node annihilations cannot be ignored as shown in Table 3. Moreover, Moore et al. [36] studied a model that has both node creations and annihilations. It was revealed that a power law degree distribution was shown to be realised in the case of a growing network with the preferential attachment rule. They also studied that the tail of the cumulative degree distribution follows a power law with an exponent *γ* = ((3 − *r*)/(1 − *r*)) − 1 > = 2.0, where *r* is a rate of node annihilations. Even though their model fits for the citation network in terms of metabolism, this study would also not match the case of the citation network because the cumulative in-degree distribution follows a power law with the exponent *γ* = 1.4 as shown in Fig 2(b).

In summary, it seems that the scale-free property of the citation network is not related to the concept of preferential attachment, which new nodes are attached to older ones with a probability which is a growing function of the number of pre-existing links [5]. A financial report (or an annual report) is written about a company’s activities throughout the following year so that a company mentions about its business partners based on their relationships which were already constructed. We, therefore, consider a fitness, which is a real number measuring companies’ importance proposed by Caldarelli et al. [26], is assigned to every firm initially. Caldarelli et al. studied scale-free networks with the fitness, and they revealed that link creations among nodes with a probability depending on the fitnesses give rise to a rich-get-richer mechanism, in which sites with more massive fitness are more likely to become hubs.

**Fig 4:**
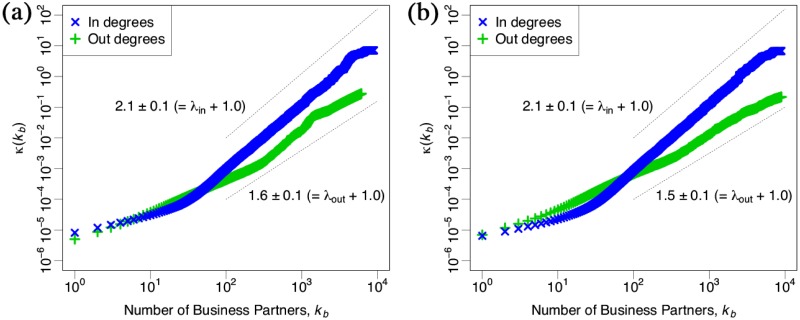
Scaling relation of the rate of fitness as a function *κ*(*k*_*b*_) of a number of business transactions *k*_*b*_ in 2018 in a log-log scale plot. Each panel (a) and (b) shows this relation of newcomers and new links on the citation network, respectively. Each red triangle and green plus-mark shows *κ*(*k*_*b*_) by out-degrees and in-degrees, respectively. Both upper and lower dotted lines indicate the power law distributions with the exponent λ_*in*_ = 1.1 ± 0.1 and λ_*out*_ = 0.6 ± 0.1, respectively.

Fig 4 shows distributions $\kappa \left(k_{b}\right)=\sum_{i=0}^{k_{b}}Q\left(i\right)/N\left(i\right)\;dk_{b}∼k_{b}^{\lambda +1}$ where *Q*(*k*_*b*_) is a probability of a new entrant connecting to an old firm of size *k*_*b*_, *N*(*k*_*b*_) is a number of firms with *k*_*b*_, and λ is an exponent of fitness for the newcomers in panel (a) and the new links in panel (b), respectively. The observation was introduced by Jeong et al. [37] though we here use the number of business transactions *k*_*b*_ which is initially given by the business transactions network so that this is the way to observe the fitness distribution. Both upper and lower dotted lines indicate power law distributions with the exponent λ_*in*_ = 1.1 ± 0.1 and λ_*out*_ = 0.6 ± 0.1, respectively.

#### Monte Carlo simulation

Here, we propose a model introducing both the fitness of each node and the effect of node annihilations as follows:

Step1 Start with *N*_0_ nodes having *m*/2 in-links and *m*/2 out-links. The end of the links is chosen randomly (*m* = 4, which is consistent with the mean number of in- and out-links for newcomers in Fig 2(c)).
Step2 Choose one of the following three events stochastically. The occurrence probabilities of link creations, link annihilations, node annihilations, and node creations are denoted by *r*_*p*_, *r*_*q*_, *r*_*r*_, and 1 − *r*_*p*_ − *r*_*q*_ − *r*_*r*_, respectively (*r*_*p*_: *r*_*q*_: *r*_*r*_ = 0.203: 0.181: 0.301, which corresponds to the observation in Table 4).
  Link creations A node is randomly selected following a rule $\Pi^{out}=\left(k_{b,i}^{\lambda_{out}}+1\right)/\sum_{j}\left(k_{b,j}^{\lambda_{out}}+1\right)$ as a starting point of a new citation link. The other end of the link is chosen randomly following a rule $\Pi^{in}=\left(k_{b,i}^{\lambda_{in}}+1\right)/\sum_{j}\left(k_{b,j}^{\lambda_{in}}+1\right)$. This process is repeated *m* times.
  Link annihilations A citation link is chosen randomly and deleted. This process is repeated *m* times.
  Node annihilations A randomly chosen node is removed along with all citation links connected to this node because a firm’s lifetime follows an exponential distribution; it is roughly consistent with the simple assumption that a firm disappears randomly following a Poisson process [18, 21].
  Node creations A new node having *m*/2 in-links and *m*/2 out-links is added. Each in- and out-link is connected to a node chosen randomly following a rule Π^*in*^ and Π^*out*^, respectively.
Step3 Repeat **Step2** for *T* times (*T* = 500, 000 in Fig 6).

**Fig 5:**
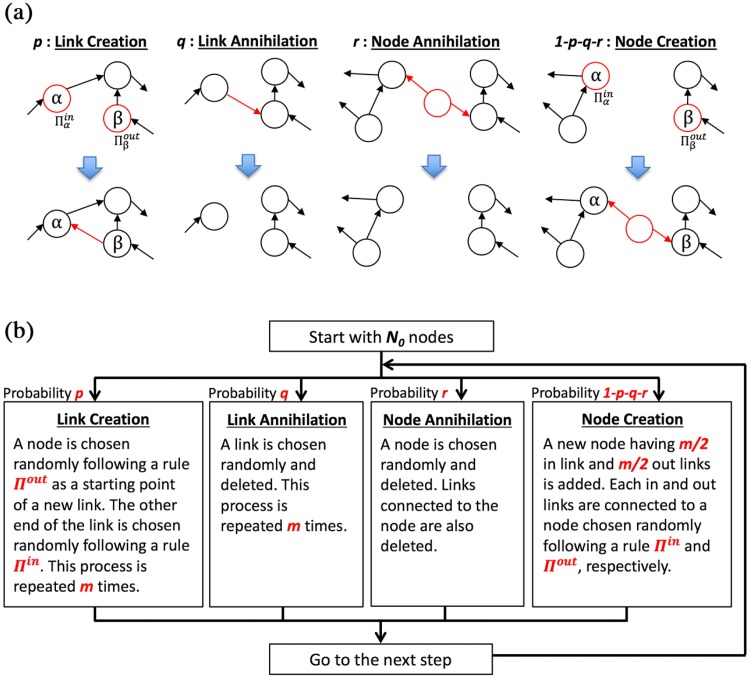
Panel (a) shows the four fundamental processes for nodes: link creations, link annihilations, node annihilations, and node creations in our simulation model. Panel (b) shows a time step flow chart of our simulation model. Each parameter set corresponds to the state of real citation network in Table 4; the occurrence probabilities *r*_*p*_: *r*_*q*_: *r*_*r*_ = 0.203: 0.181: 0.301, each the preferential attachment exponent for in-links and out-links is λ_*in*_ = 1.0 and λ_*out*_ = 0.5, respectively.

We also illustrate our algorithm by a flow chart in Fig 5.

**Fig 6:**
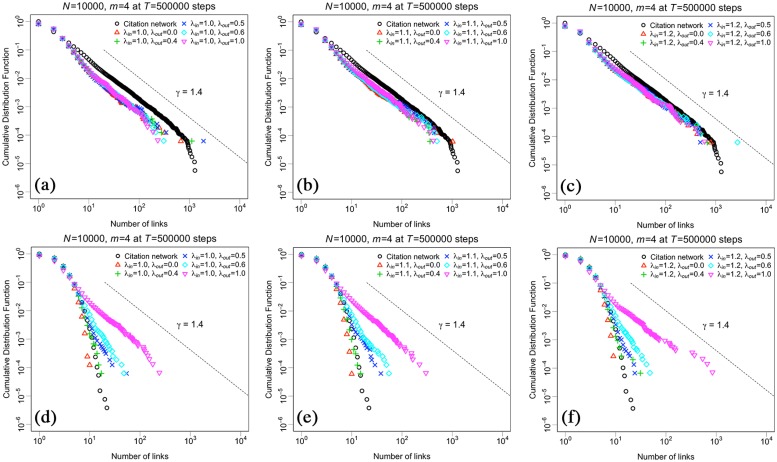
Cumulative degree distributions generated by the simulation results in a log-log scale plot by *N* = 10, 000, *T* = 500, 000, and *m* = 4. Each panel (a), (b) and (c) shows the in-degree distribution by λ_*in*_ = 1.0, λ_*in*_ = 1.1 and λ_*in*_ = 1.2, respectively, and each panel (d), (e) and (f) shows the out-degree distribution by λ_*in*_ = 1.0, λ_*in*_ = 1.1 and λ_*in*_ = 1.2, respectively. Each black circle, red triangle, green plus-mark, blue cross, light-blue rhombus, and pink descending triangle show the result by the citation network in 2018, λ_*out*_ = 0.0, λ_*out*_ = 0.4, λ_*out*_ = 0.5, λ_*out*_ = 0.6, and λ_*out*_ = 1.0, respectively. Each dotted line indicates the power law distribution with the exponent *γ* = 1.4.

In order to verify whether our model grasps the essence of dynamics of the citation network, we compare both degree distributions and motif formation distributions generated by the simulation results using various parameters with the observations in Figs 2 and 3. Fig 6 shows the cumulative degree distributions in a log-log scale plot by *N* = 10, 000, *T* = 500, 000, and *m* = 4, which is roughly consistent with the mean number of in- and out-links for newcomers in Fig 2(c). Each panel (a), (b) and (c) shows in-degree distributions by λ_*in*_ = 1.0, λ_*in*_ = 1.1 and λ_*in*_ = 1.2, respectively, and each panel (d), (e) and (f) shows out-degree distributions by λ_*in*_ = 1.0, λ_*in*_ = 1.1 and λ_*in*_ = 1.2, respectively. Each black circle, red triangle, green plus-mark, blue cross, light-blue rhombus, and pink descending triangle show the result by the citation network in 2018, λ_*out*_ = 0.0, λ_*out*_ = 0.4, λ_*out*_ = 0.5, λ_*out*_ = 0.6, and λ_*out*_ = 1.0, respectively. Each dotted line indicates power law distributions with the exponent *γ* = 1.4 the same as Fig 2. Regarding the in-degree distribution, the results by λ_*in*_ = 1.1 ∼ 1.2 fit well with the power law distribution with the exponent *γ* = 1.4. Moreover, the simulation results by λ_*out*_ = 0.5 ± 0.1 also work to replicate exponentially decays of the out-degree distribution. These parameters correspond with the observation in Fig 4; therefore, we confirm the model seems to be reasonable in terms of distributions of degree.

**Fig 7:**
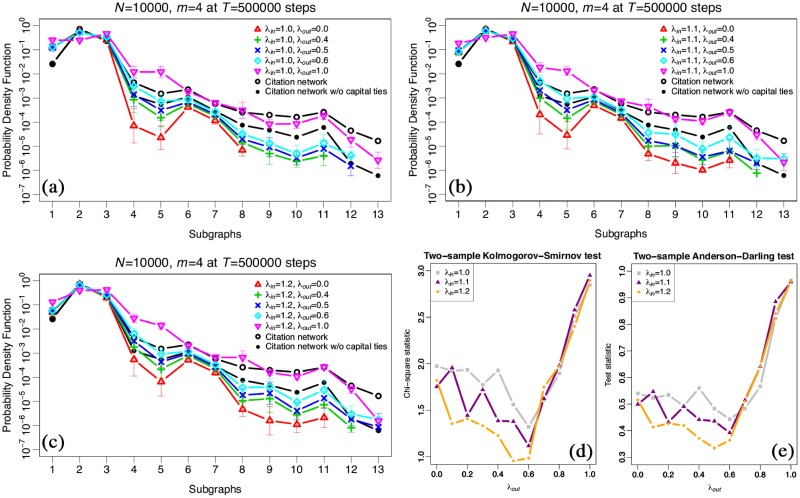
Probability density distributions of subgraphs of network motif generated by the simulation results in a semilog scale plot by *N* = 10, 000, *T* = 500, 000, and *m* = 4. Each panel (a), (b) and (c) shows the distribution by λ_*in*_ = 1.0, λ_*in*_ = 1.1 and λ_*in*_ = 1.2, respectively. Each red triangle, green plus-mark, blue cross, light-blue rhombus, pink descending triangle, black circle, and black filled circle show the result by λ_*out*_ = 0.0, λ_*out*_ = 0.4, λ_*out*_ = 0.5, λ_*out*_ = 0.6, λ_*out*_ = 1.0, the citation network in 2018, and the citation network without capital ties, respectively. Each panel (d) and (e) shows the results of the two-sample Kolmogorov–Smirnov test and Anderson–Darling test between real data and the simulation results, respectively. Both horizontal lines show λ_*out*_, and each vertical line shows the chi-square statistic and the test statistic as defined above, respectively. Each grey filled square, purple filled triangle, and orange filled rhombus shows λ_*in*_ = 1.0, λ_*in*_ = 1.1, and λ_*in*_ = 1.2, respectively.

Fig 7 shows probability density distributions of subgraphs of network motif in a semilog scale plot by the same simulation results as Fig 6. Each panel (a), (b) and (c) shows the distributions by λ_*in*_ = 1.0, λ_*in*_ = 1.1 and λ_*in*_ = 1.2, respectively. Each red triangle, green plus-mark, blue cross, light-blue rhombus, pink descending triangle, black circle, and black filled circle show the result by λ_*out*_ = 0.0, λ_*out*_ = 0.4, λ_*out*_ = 0.5, λ_*out*_ = 0.6, λ_*out*_ = 1.0, the citation network in 2018, and the citation network without capital ties, respectively. By comparison these simulation results with the distribution of the citation network, our model does not fit well. Similar to the comparison results of the real network with the randomised network as shown in Fig 3, there are especially differences in frequencies of subgraphs that have bidirectional links.

For this reason, we investigate bidirectional links on the citation network. As shown in Table 2, there are 587,213 links. Among these links, 568,901 pairs of firms (about 97% of all links) are uni-directional, and the rest of 9,156 pairs of firms (about 3%) are bidirectional. To understand the meaning of each link, we check overlapping among links on the citation network, list of inter-firm business transactions and list of inter-firm investment relationships. Table 5 shows the details. It appears that about 76% of bidirectional links on the citation network overlap links on the list of inter-firm investment relationships, though about only 10% of unidirectional links overlap links on that. We simultaneously attempt to delete overlapping links on the citation network with links on the list of inter-firm investment relationships (hereafter referred to as “capital ties”) and compare our simulation results with the probability density distribution of subgraphs on the network (black filled circle in Fig 7). It appears that our results work better to replicate the motif formation distribution on the citation network with capital ties excluded from the citation network. Therefore, it is confirmed that our model reflects the dynamics of firms’ interactions, mainly inter-firm business transactions, on the citation network without the real frequency of bidirectional links that are mainly generated by inter-firm investment relationships and a tiny component of the network. This is consistent with the fact that in any group structures both parent companies as well as subsidiaries must report the relationship [38], and therefore, leading to bidirectional links.

**Table 5:** Number of overlapping links among links on the citation network, list of inter-firm business transactions and list of inter-firm investment relationships.

Unidirectional links on Citation network	Overlap with list of Business transactions	Overlap with list of Investment relationships	Overlap with both lists	Non Overlapping	Total links
2017	271,402	17,029	40,711	229,295	558,437
2018	289,002	16,560	41,888	221,451	568,901
Bidirectional links on Citation network	Overlap with Business transactions	Overlap with Investment relationships	Overlap with both lists	Non Overlapping	Total links
2017	1,559	1,322	5,471	482	8,834
2018	1,771	1,296	5,673	416	9,156

Furthermore, to find the best parameter to replicate the motif formation distribution, we apply both the two-sample Kolmogorov–Smirnov test [39] and the two-sample Anderson–Darling test [40], which measure the difference between two distributions. Anderson–Darling test gives more weight to the tails of the distribution, whereas Kolmogorov-Smirnov test is more sensitive of the center of distribution [41, 42]. The definition of chi-square statistic in the Kolmogorov–Smirnov test *χ*^2^ = 4*D*^2^ ⋅ *mn*/(*m* + *n*), where test statistic *D* is a maximum vertical deviation between two distributions and *m* and *n* are the number of samples of those distributions. Moreover, the definition of the two-sample Anderson–Darling test statistic is as follows:

$$\begin{array}{c} AD=\frac{mn}{m+n}\int_{-\infty }^{\infty }\frac{{\left\{F_{m}\left(x\right)-G_{n}\left(x\right)\right\}}^{2}}{H_{m+n}\left(x\right)\{1-H_{m+n}\left(x\right)\}}dH_{m+n}\left(x\right), \end{array}$$ (1)

where each *F*_*m*_(*x*) and *G*_*n*_(*x*) is the empirical distribution functions of the sample and *H*_*m*+*n*_(*x*) = {*mF*_*m*_(*x*) + *nG*_*n*_(*x*)}/(*m* + *n*) is the empirical distribution function of the pooled sample [40]. Fig 7(d) and 7(e) show the results for each parameter. Each horizontal line and vertical line shows λ_*out*_ and test statistics as defined above, respectively, and each grey filled square, purple filled triangle and orange filled rhombus shows λ_*in*_ = 1.0, λ_*in*_ = 1.1, and λ_*in*_ = 1.2, respectively. As a result of this, we find that the simulated distribution comes closest to the real one with the parameter set λ_*in*_ = 1.2, λ_*out*_ = 0.5 which fits well with the real observation.

## Conclusions

In this paper, we investigated firms’ interactions generated by automated scanning of financial reports of around 400, 000 firms in Japan, in order to establish the essential dynamics of interactions between firms. As key finding, we observe that the metabolism of the derived citation network is significantly different from that of the actual inter-firm business transactions network that had previously been studied. Remarkably, we found that about quarter of links on the citation network were exchanged for next year so that the rewiring was one of the dominant processes in the network, even though only about 16.7% links on the inter-firm business transactions network exchanged for next year. Furthermore, we introduced simulations based on a network evolution model with fitness because the financial reports were written about a firms’ activities throughout the following year so that firms mentioned about its business partners based on their relationships which were already constructed. Our results show that our model is able to replicate the statistical properties of the citation network with parameters estimated from real underlying data. It is also found that the bidirectional links, which overlapped with the inter-firm investment relationships, was hard to replicate by our simple numerical model. Even though the bidirectional links were tiny components of the citation network, this result suggested that essential investment interactions tended to be generated through a divergent process from that of the business transactions.
